# Valorisation Potential of Invasive *Acacia dealbata*, *A. longifolia* and *A. melanoxylon* from Land Clearings

**DOI:** 10.3390/molecules27207006

**Published:** 2022-10-18

**Authors:** Ricardo M. F. da Costa, Maurice Bosch, Rachael Simister, Leonardo D. Gomez, Jorge M. Canhoto, Luís A. E. Batista de Carvalho

**Affiliations:** 1 Molecular Physical-Chemistry R&D Unit, Department of Chemistry, University of Coimbra, Rua Larga, 3004-535 Coimbra, Portugal; 2 Centre for Functional Ecology, Department of Life Sciences, University of Coimbra, Calçada Martim de Freitas, 3000-456 Coimbra, Portugal; 3 Institute of Biological, Environmental and Rural Sciences, Aberystwyth University, Plas Gogerddan, Aberystwyth, Ceredigion SY23 3EE, UK; 4 Centre for Novel Agricultural Products, Department of Biology, University of York, Heslington, York YO10 5DD, UK

**Keywords:** *Acacia*, biomass, cell wall, white-rot fungi, wildfire

## Abstract

*Acacia* spp. are invasive in Southern Europe, and their high propagation rates produce excessive biomass, exacerbating wildfire risk. However, lignocellulosic biomass from *Acacia* spp. may be utilised for diverse biorefinery applications. In this study, attenuated total reflectance Fourier transform infrared spectroscopy (FTIR-ATR), high-performance anion-exchange chromatography pulsed amperometric detection (HPAEC-PAD) and lignin content determinations were used for a comparative compositional characterisation of *A*. *dealbata*, *A*. *longifolia* and *A*. *melanoxylon*. Additionally, biomass was treated with three white-rot fungi species (*Ganoderma lucidum*, *Pleurotus ostreatus* and *Trametes versicolor*), which preferentially degrade lignin. Our results showed that the pre-treatments do not significantly alter neutral sugar composition while reducing lignin content. Sugar release from enzymatic saccharification was enhanced, in some cases possibly due to a synergy between white-rot fungi and mild alkali pretreatments. For example, in *A*. *dealbata* stems treated with alkali and *P*. *ostreatus*, saccharification yield was 702.3 nmol mg^−1^, which is higher than the samples treated only with alkali (608.1 nmol mg^−1^), and 2.9-fold higher than the non-pretreated controls (243.9 nmol mg^−1^). By characterising biomass and pretreatments, generated data creates value for unused biomass resources, contributing to the implementation of sustainable biorefining systems. In due course, the generated value will lead to economic incentives for landowners to cut back invasive *Acacia* spp. more frequently, thus reducing excess biomass, which exacerbates wildfire risk.

## 1. Introduction

Trees and shrubs of the *Acacia* genus originate in Australia and belong to the Fabaceae, subfamily Mimosoideae, comprising more than 1350 species [1]. In Europe, particularly in Portugal, *Acacia dealbata* Link, *Acacia longifolia* (Andrews) Willd, and *Acacia melanoxylon* R.Br. are considered invasive species, having been brought as ornamentals to stabilise dunes and for timber and tanning applications [2]. These *Acacia* are highly adaptable to a wide range of soils with low fertility and have high growth rates, thus reducing the development of autochthonous species. Furthermore, they produce high amounts of biomass with high latent combustibility, which exacerbates wildfires. For this reason, there have been several studies focusing on the potential of *Acacia* spp. biomass for various applications [3,4], or the use of these species for the capture and sequestration of carbon [5], while reducing their impact on wildfires in Portugal [6].

Lignin content is positively correlated with the heat of combustion of wood [7]. Therefore, abundant lignin in the environment increases the risk of wildfires. Nonetheless, the high amounts of lignocellulosic biomass contained in these species can be used for various fermentation-based applications, from cellulosic ethanol [8] to biogas [9], or even for the production of lactic [10], citric [11], acetic [12], and succinic acids [13], via lignocellulose fermentation. Alternatively, there is also a range of products that may be derived from hemicelluloses and lignin fractions. A few examples include the use of pentoses in hemicellulose hydrolysates for ethanol fermentation [14], xylitol and xylonate production via fermentation of xylose [15], or the use of lignin building blocks for the production of plastics, industrial additives or biomedical applications [16].

The present work focused mainly on the conversion of *Acacia* spp. cell wall glycans into fermentable sugars via enzymatic saccharification. Lignocellulosic biomass is highly recalcitrant to enzymatic deconstruction; hence pretreatments are usually employed to reduce recalcitrance. Pretreatments have the purpose of altering the biomass structure by disrupting their inter-unit covalent and non-covalent interactions [17]. Alkaline pretreatments have been considered particularly promising procedures to increase the biodegradability of lignocellulosic feedstocks [18]. In contrast to acid pretreatments, mild alkali does not cause a significant release of individual monosaccharide components, as these pretreatments primarily lead to solvation and saponification of the cell wall, thereby making cellulose more accessible to hydrolytic enzymes [19,20,21,22]. Mild alkali pretreatment has been proven to significantly increase enzymatic saccharification yields [23,24], and consequently, we employed it here.

Biological pretreatments, namely by fungi, are another promising biomass pretreatment method [25,26]. Here, we hypothesise that biological pretreatment of wood with white-rot fungi (WRF) could significantly improve the results obtained by a mild alkali pretreatment. The capacity of WRF to selectively degrade lignin without extensive cellulose degradation is a characteristic that makes them ideally suited for biodelignification applications, where lignin or various phenolic compounds must be altered or removed [27,28]. WRF have developed ligninolytic enzymatic machinery, including a wide range of peroxidases and laccases [29,30,31]. These diverse enzymatic pools allow WRF to deal with different compositional and structural aspects and depolymerise lignin. In the wild, WRF might have a beneficial effect on wildfire prevention, as they reduce lignin content and thus reduce the calorific value of the biomass. From a biorefinery perspective, biological pretreatments have been less considered than thermochemical ones, possibly because the industry often finds slower processing rates unattractive. However, this problem can be addressed by continuous flow processing systems [32]. Furthermore, although compared to other pretreatments, WRF involve longer processes, the energy requirements are low, and treatment conditions are mild and more environment-friendly [33,34]. In this study, we aim to evaluate a bio-alkali pretreatment process (fungal pretreatment combined with 0.1 M NaOH) for the bioconversion of *Acacia dealbata*, *A*. *longifolia* and *A*. *melanoxylon* into fermentable sugars. The studied *Acacia* spp. have high fire tolerance, and their spread is promoted in post-fire conditions. Creating applications for acacia biomass will generate an economic incentive for landowners to more frequently cut back these invasive species, thus preventing wildfires and reducing their severity.

## 2. Results

### 2.1. Acacia Cell Wall Compositional Characterisation and Pretreatment Effects

**Figure 1:**
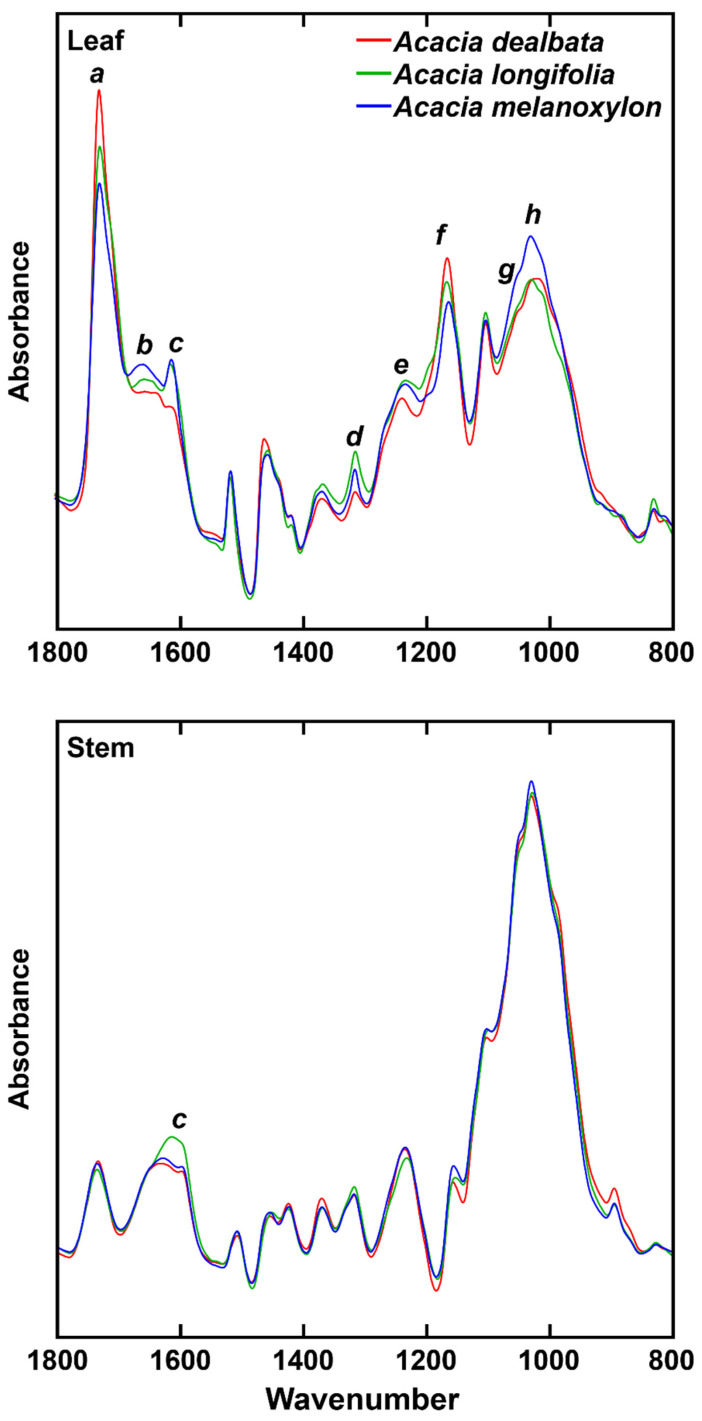
Mean FTIR-ATR spectra of leaf and stem samples of three *Acacia* spp. in the range 1800–800 cm^−1^. Spectral bands: *a*, 1728 cm^−1^; *b*, 1640 cm^−1^; *c*, 1612 cm^−1^; *d*, 1314 cm^−1^; *e*, 1238 cm^−1^; *f*, 1166 cm^−1^; *g*, 1055 cm^−1^; *h*, 1030 cm^−1^.

FTIR-ATR was used for the characterisation of alcohol-insoluble residues (AIR) from *A. dealbata*, *A*. *longifolia* and *A*. *melanoxylon*. The most noticeable distinct spectral regions between the species are highlighted (Figure 1). For leaf and stem biomass, eight distinct regions were identified, centred at (cm^−1^): 1728 (*a*), 1640 (*b*), 1612 (*c*), 1314 (*d*), 1238 (*e*), 1166 (*f*), 1055 (*g*) and 1030 (*h*) (Table 1).

There was a predominance of bands ascribed to polysaccharides, with spectral regions *a* and *e* having been assigned to xylan structural features (Table 1). Bands *g* and *h* have been attributed to vibration in cellulose. Regarding lignin, band *c* was ascribed to aromatic skeletal vibrations and *d* was assigned to syringyl monomer vibrations. The spectral data show that leaf biomass has a more divergent composition than stem biomass in *Acacia* spp. For the latter, the most prominent difference was higher intensities of lignin signals (band *c*, 1612 cm^−1^) in *A*. *longifolia*.

**Figure 2:**
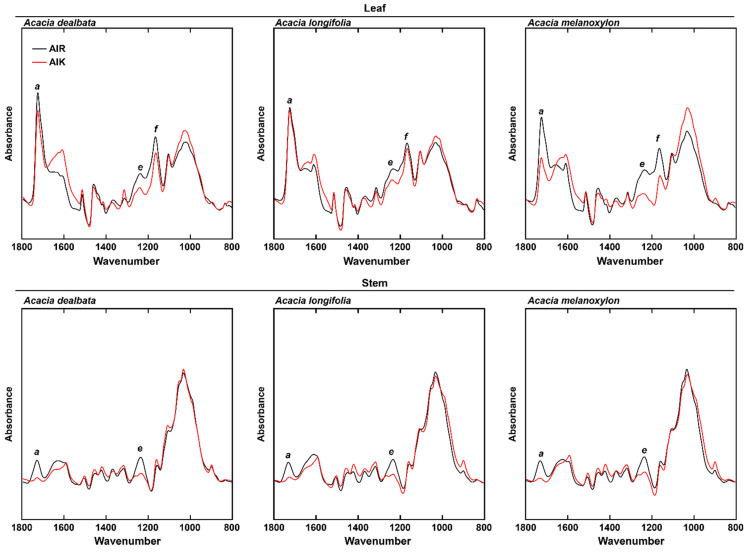
Mean FTIR-ATR spectra of non-pretreated alcohol insoluble residue (AIR) and mild alkali-pretreated alcohol insoluble residue (AIK) *Acacia* spp. biomass. Spectral bands: *a*, 1728 cm^−1^; *e*, 1238 cm^−1^; *f*, 1166 cm^−1^.

To assess the effect of a mild alkali pretreatment on the cell wall from *A. dealbata*, *A*. *longifolia,* and *A*. *melanoxylon*, AIR samples were treated with 0.1 M NaOH for 24 h at 21 °C (AIK). FTIR-ATR was subsequently employed to assess the main compositional changes effected by the alkali. For stem, of the three examined species, pretreated biomass showed a reduction in the *a* (1728 cm^−1^) and *e* (1238 cm^−1^) spectral regions (Figure 2). For leaf, a reduction in the intensities was additionally observed in region *f* (1166 cm^−1^). Band *a* was assigned to C=O stretching in xylans, band *e* is associated with C-O vibrations of acetyl, and band *f* was ascribed to glycosidic stretching in polysaccharides.

**Figure 3:**
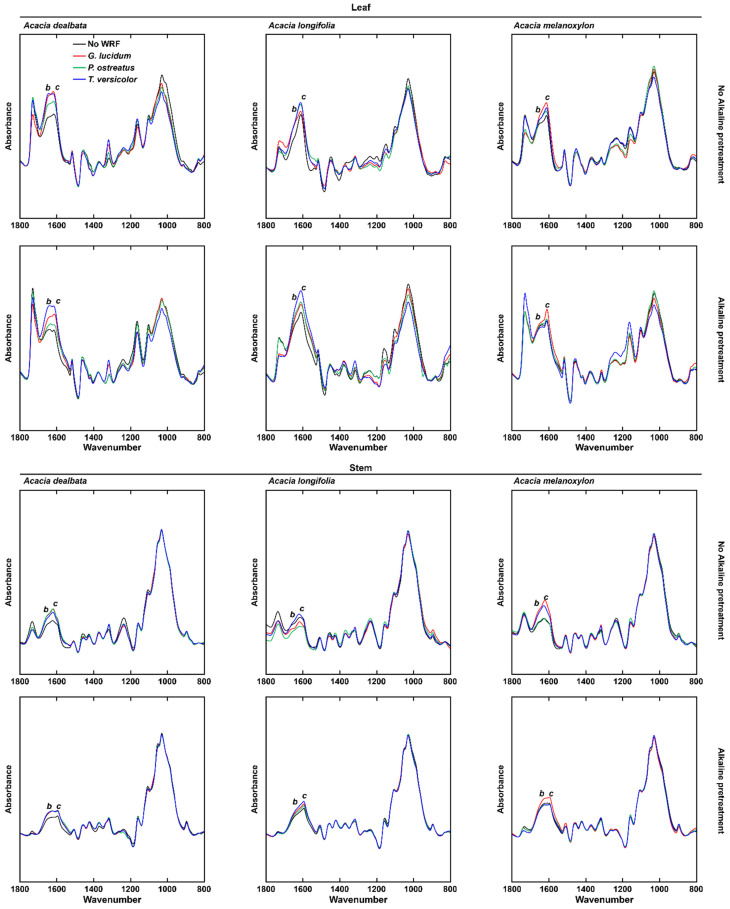
Mean FTIR-ATR spectra of milled but not organic solvent-washed biomass samples from *Acacia* spp., treated with three white-rot fungi species (WRF; 30 days) and/or in combination with an alkali pretreatment. Spectral bands: *b*, 1640 cm^−1^; *c*, 1612 cm^−1^.

In addition to the mild-alkali pretreatment, the samples were pretreated with white-rot fungi (WRF). To assess the effect of these pretreatments on composition, FTIR-ATR was employed (Figure 3). Interestingly, the effect of a given WRF species varies between different biomass species. Similarly, the same biomass is differently affected by each WRF species. In leaf treated only with WRF, most of the variation was observed in the *b* and *c* spectral regions, where both showed higher intensities in relation to the non-WRF treated (NF) control samples. In stems, the most affected regions when only WRF pretreatment was employed also are *b* and *c* bands, showing higher intensities than NF, particularly in *A*. *dealbata* and *A*. *melanoxylon*. When the combined WRF-ALK pretreatments were employed, the effect of the fungi is once again seen in regions *b* and *c* of the spectra of leaves. For stems, the WRF-ALK-treated samples were less altered in relation to the biomass treated only with the mild alkali pretreatment.

### 2.2. Saccharification Yields of Acacia Biomass

**Figure 4:**
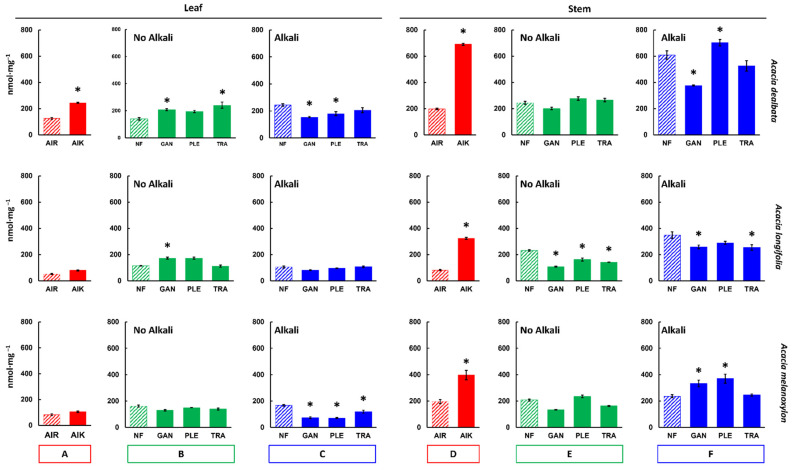
Saccharification of *Acacia* spp. biomass. Mean nmol of reducing sugar released per mg of biomass material (nmol mg^−1^) after 8 h incubation in a hydrolytic enzyme mixture. Red bars (**A**,**D**) refer to non-pretreated alcohol insoluble residue samples, without (AIR) or with (AIK), a mild alkali pretreatment. Green bars (**B**,**E**) refer to samples treated only with white rot fungi (WRF): *G. lucidum*, GAN; *P. ostreatus*, PLE; *T. versicolor*, TRA. Blue bars (**C**,**F**) designate treatments where the alkali treatment was employed subsequently to the WRF pretreatment. Pairwise *t*-tests were performed between each treatment and non-pretreated control samples (AIR; or NF, no fungi; striped bars) to evaluate the impact of the pretreatments. The treatments which are significantly different from the controls are marked with a “*” (*p* ≤ 0.05). Error bars represent the standard error of the sample replicates.

An enzymatic saccharification assay was performed on all samples to compare biorefining potentials between the different acacia species and to investigate the effect of the applied pretreatments. AIR prepared from *A. dealbata* biomass showed the highest saccharification yields in leaves and in stems: 125.5 and 197.9 nmol mg^−1^ after 8 h incubation, respectively (Figure 4 and Table 2). The AIR samples were also treated with a mild alkali pretreatment (AIK) 0.1 M NaOH (24 h at 21 °C). In these samples, *A. dealbata* also showed the highest saccharification yield in leaf and stem samples: 243.3 nmol mg^−1^ and 690.0 nmol mg^−1^. Saccharification yields were typically higher from stems than leaves.

For each of the applied pretreatments, the percentage of recovered solids was calculated (Supplementary Table S1) for each species and organ. From WRF-treated leaf samples, typically 94% of the biomass is recovered after a 30-day incubation. In stems, the value is 96% for the same conditions. When the 0.1 M NaOH mild alkali pretreatment alone is employed, 93% of leaf and 96% of stem biomass is recovered. As for combined WRF-ALK pretreatments, recovered percentages drop to 90% from leaves and 94% from stems.

Biomass treated with WRF showed increased saccharification yields under selected conditions when compared with NF controls (Figure 4B,E; green bars). In leaves, the highest saccharification yield from samples treated with WRF alone was observed using *T*. *versicolor* on *A*. *dealbata* biomass (240.8 nmol mg^−1^; Figure 4), 72.0% higher than NF controls (*p* ≤ 0.05; Figure 4). For stems, the highest value was 276.9 nmol mg^−1^ obtained from treating *A*. *dealbata* with *P*. *ostreatus*.

A mild alkali pretreatment (0.1 M NaOH; 24 h; 21 °C) was also employed in sequence with the WRF pretreatment (Figure 4C,F; blue bars) to extract compounds which may have a negative effect on saccharification. This alkaline treatment substantially increased saccharification yields. The highest, significantly different (*p ≤* 0.05) saccharification yield in relation to the controls was seen with *A*. *dealbata* stems treated with *P*. *ostreatus* and 0.1 M NaOH (702.3 nmol mg^−1^; Figure 4 blue bars).

Given that most of the highest saccharification yields were obtained from biomasses treated with *P*. *ostreatus*, subsequent cell wall and pretreatment liquid fraction compositional analyses were performed only for samples treated with this ligninolytic fungal species.

### 2.3. Acacia Cell Wall Neutral Sugars and Lignin Composition

**Figure 5:**
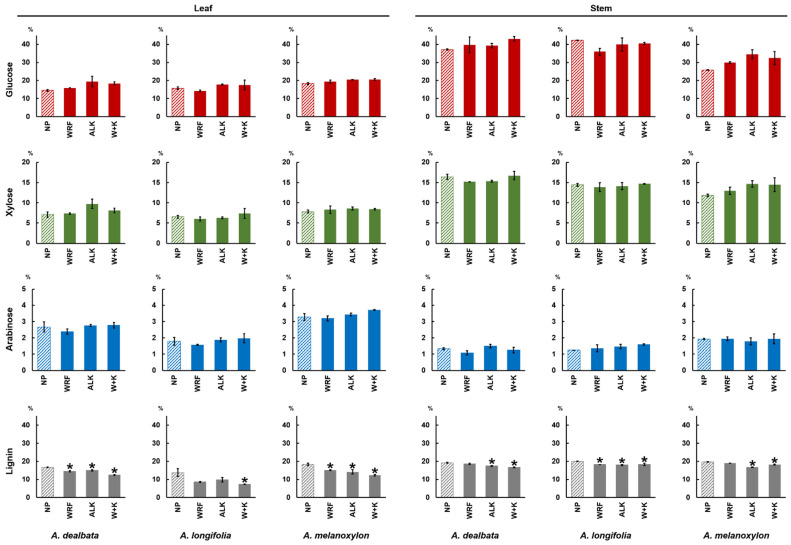
Mean percentage (%) composition of alcohol insoluble residues (AIR) prepared from pretreated biomass from three *Acacia* spp. Pretreatment acronyms: NP, non-pretreated control samples (striped bars); WRF, samples pretreated with *P. ostreatus*; ALK, alkali-pretreated samples; W + K, samples pretreated with *P*. *ostreatus* followed by alkali pretreatment. Pairwise *t*-tests were performed between each treatment and NP samples to evaluate the impact of the pretreatments; the treatments which are significantly different from the NP controls are marked with a “*” (*p* ≤ 0.05). Error bars represent the standard error of the sample replicates.

The major cell wall neutral sugars and lignin contents were determined for leaf and stem samples from the acacia species examined in this study. In non-pretreated control samples, the biomass from leaves contained (% AIR dry weight, DW): 14.5% glucose, 7.1% xylose, 2.7% arabinose and 16.7% lignin for *A*. *dealbata*; 15.8% glucose, 6.6% xylose, 1.8% arabinose and 13.8% lignin for *A*. *longifolia*; 18.3% glucose, 7.8% xylose, 3.3% arabinose, and 18.3% lignin for *A*. *melanoxylon* (Figure 5 and Table 3). For these four parameters measured in leaves, the values were typically higher in *A. melanoxylon*. In stem biomass, the corresponding values were: 37.1% glucose, 16.4% xylose, 1.3% arabinose, and 19.2% lignin for *A. dealbata*; 42.3% glucose, 14.5% xylose, 1.2% arabinose and 20.0% lignin for *A. longifolia*; 25.7% glucose, 11.8% xylose, 1.9% arabinose, and 19.7% lignin for *A. melanoxylon* (Table 3). Highest glucose and lignin content was found in *A. longifolia*, whereas the highest xylose was in *A. dealbata* and arabinose in *A. melanoxylon*.

In biomass treated with WRF, 0.1 M NaOH mild alkali or a combination of both, in most cases, neutral sugar composition did not change significantly in relation to the non-pretreated samples (*p* > 0.05). Despite not being significantly different, in some cases, samples with WRF or ALK showed slightly higher amounts of neutral sugars compared with non-pretreated biomass. These slight increases, particularly in alkali-pretreated samples, are likely to be related to the pretreatment effect, which improved acid hydrolysis efficiency. By contrast, statistically significant differences for lignin were found (*p ≤* 0.05; Figure 5). There was a decrease in lignin content in relation to non-pretreated samples. These results demonstrate that the action of the employed pretreatments produces a decrease in lignin content without significantly affecting neutral sugar composition.

## 3. Discussion

### 3.1. Characterisation of the Biomass from Acacia Species

Acacia biomass has been considered for various applications, such as pulp manufacture, sawed wood or furniture. It may also be used as a raw material to obtain sugars and sugar oligomers from its polysaccharide components, namely from xylan, as acacias may be an interesting source of xylooligosaccharides [52]. A variety of analytical techniques was employed to determine the biomass composition and biorefining potential of *A*. *dealbata*, *A*. *longifolia,* and *A*. *melanoxylon* from marginal lands in Portugal. Leaf and stem were analysed separately in the present work instead of pooled total above-ground biomass, as each of these types of biomass have very distinct properties in a biorefinery context, namely because leaves are, to a large extent, composed of cells lacking secondary cell walls, which means less lignin content.

Alcohol insoluble residues (AIR) were prepared from non-pretreated biomass samples from leaf and stem from the acacia species under study. From this material, neutral sugars, and acetyl bromide soluble lignin contents were determined. The main neutral monosaccharides in all three acacia species were glucose, xylose, and arabinose, with a respectively overall mean of 16.2%, 7.2% and 2.6% for leaves, and 35.0%, 14.2% and 1.5% for stems. These values agree with data reported elsewhere for acacia stems [53,54]. As for leaves, no reports could be found in the literature about the composition of this organ’s biomass in these acacia species. Therefore, the present work may be the first to report such values. In all species, glucose and xylose content was higher in stems than in leaves, whereas for arabinose, the amounts were higher in leaves (Figure 5 and Table 3). Lignin was determined using the acetyl bromide method, showing an overall mean of 16.3% for leaves and 19.6% for stems of the studied species. Once again, these values agree with those reported elsewhere for the acacia stems [53,54].

FTIR-ATR analysis showed that in stems, the main difference between species is in band *c* (1612 cm^−1^), which has been ascribed to aromatic skeletal vibration. FTIR-ATR results also suggest that the wood from these three acacia species is less variable than leaf biomass. In leaves, presumably as a result of distinct leaf physiognomy between the species, there is more variability among the spectra and, thus, their composition. In stems, by contrast, despite the differences seen in glucose, xylose and arabinose, lignin content does not vary much between the species. Given the similarity among the wood of the different species, they can be processed using similar biorefining processes.

### 3.2. Mild Alkali Pretreatment and Impact on Saccharification

Mild alkali pretreatments have the potential to cause de-esterification in the biomass, minimising lignin and carbohydrate losses [55,56,57], resulting in increased biodegradability of lignocellulosic feedstocks [58,59]. To further understand the potential of the studied acacia biomasses for applications in biorefining, AIR samples were treated with a mild alkali pretreatment followed by enzymatic saccharification. The alkali pretreatment had a significant effect on saccharification (*p* ≤ 0.05; Figure 4), as AIK yields were, on average, 2.4-fold higher than AIR., being 1.6-fold for leaves, and 3.2-fold for stems. However, the increase also varied between the species (Table 2). The most substantial effect of the alkali pretreatment was seen in *A. longifolia*, as AIK yields were 4-fold higher in stems treated with a mild alkali. *A. dealbata* stems showed an increase in saccharification around 3.5-fold in AIK yields.

FTIR-ATR analysis (Figure 2) showed differences between AIR and AIK samples, particularly at spectral regions *a* (1728 cm^−1^; assigned to C=O stretching in acetyl-xylans) and *e* (1238 cm^−1^; attributed to C-O vibrations of acetyl) (Table 1). The intensity of these bands is reduced in pretreated biomass and is observed across all species. Nonetheless, the deacetylation effect appears to be more complete in stem biomass than in leaves. This is presumably due to the presence of higher amounts of secondary metabolites in leaves, given their more diversified physiological roles when compared to stems. For other lignocellulosic biomasses, it has been reported that alkaline saponification during mild alkali pretreatment can release acetyl ester substituents from heteroxylans [60]. This concurs with our results that the most noticeable and consistent compositional differences between AIR and AIK samples are seen with bands associated with xylan and acetyl substituents. These differences are the result of the loss of acetyl groups in the biomass, specifically in xylans, as *O*-acetylated xylan is the main source of acetylesters in lignocellulosic biomass [61].

### 3.3. Effects of White-Rot Fungi Pretreatments in Acacia Biomass

Polysaccharide hydrolysis is enhanced by the formation of pores in the plant cell wall during biodelignification, increasing saccharification yields [62]. As their hyphae penetrate the biomass, WRF degrades lignin more readily than holocellulose [31,63,64,65], and this trait was assessed by pretreating acacia biomass with these fungi. By comparing the FTIR-ATR spectra of stem biomass treated with WRF with the spectra from NF controls, we observed that stems are less affected by the fungal pretreatment than leaves (Figure 4). According to the modifications in the FTIR-ATR spectra of leaves, it is in *A*. *dealbata* that the most noticeable effect of the WRF treatment is observed, particularly in spectral regions *b* (1640 cm^−1^) and *c* (1612 cm^−1^). These regions have been correlated with non-esterified carboxyl groups in polysaccharides and aromatic skeletal vibration (Table 1). This may result from WRF-mediated de-esterification and modification of ester-linked phenolic hydroxycinnamates involved in cross-linking of cell wall polymers, including lignin [66,67].

In the saccharification assay performed in this study, an increase in saccharification yields in relation to the NF controls was observed in leaf biomass from *A. dealbata* treated with the three fungal species, and in *A. longifolia* treated with *G*. *lucidum* and *P*. *ostreatus*. For stems, yield increases were seen with *A. dealbata* treated with *P. ostreatus*, *T. versicolor*, and with *A. melanoxylon* treated with *P. ostreatus*. However, in other cases, when samples were treated with WRF, the saccharification yields were lower than the controls (Figure 4 and Table 2). This is presumably due to the release of enzyme-inhibitory compounds during fungal action. It is known that pretreatment-derived soluble compounds can inhibit saccharification by binding the hydrolytic enzymes [68,69,70]. To address this issue and enhance pretreatment efficiency, a mild alkali pretreatment was employed (0.1 M NaOH; 24 h; 21 °C), which substantially increased saccharification yields in relation to non-alkali treated samples. Our results suggest that a synergistic effect does occur when the WRF and mild alkali pretreatments are combined. In *A. dealbata* stems treated with alkali and *P. ostreatus*, the saccharification yield was 702.3 nmol mg^−1^, which is higher than the samples treated only with alkali (608.1 nmol mg^−1^), and 2.9-fold higher than the non-pretreated controls (243.9 nmol mg^−1^) (Figure 4 and Table 2). Furthermore, lignin content in the pretreated samples is lower than in non-pretreated controls (Table 3 and Figure 5). It is likely that some lignin loss or structural alteration is responsible for the increase seen in saccharification yield. These modifications are likely to be in cell wall polymers, such as lignin, and ester-bound substituents, such as ferulic acid. A structurally compromised cell wall would be more susceptible to hydrolysis by cellulolytic enzymes, leading to increased saccharification yields.

## 4. Materials and Methods

### 4.1. Acacia Lignocellulosic Biomass

Biomass from wild-grown *Acacia dealbata* (ACD), *A*. *longifolia* (ACL) and *A*. *melanoxylon* (ACM) was collected at a location in Central Portugal, Serra da Boa Viagem (40.186115° N, 8.903903° W) replicating the biomass that would result from biomass clearings. Sampling was performed during late summer (September). All samples consisted of whole branches cut at trunk level from non-senesced, field-growing plants. For each species, two biological replicates were collected, discarding any seeds, fruits, or flowers, when present. Within a maximum of 5 h from the collection, all samples were stored at −80 °C until freeze-drying. Once dry, stems were separated from leaves, and individual organs were ground using a Retsch SR3 Rotor Beater Mill (Retsch, Haan, Germany) and passed through a perforated plate screen with 2 mm diameter holes.

### 4.2. Preparation of Alcohol Insoluble Residue

A procedure based on a protocol reported by da Costa et al. 2020 [71] was carried out to produce the alcohol-insoluble residue (AIR) used in the subsequent analyses. For each sample, approximately 1 g of ground plant biomass was extracted sequentially as follows: 30 mL 70% (*v*/*v*) aqueous ethanol (first for 12 h and then twice for 30 min in a shaking incubator set at 40 °C/150 rpm), three times with 20 mL chloroform/methanol (1:1 *v*/*v*) (30 min incubation at 25 °C and 150 rpm), and finally, three times with 15 mL acetone (30 min, at 25 °C/150 rpm). Between each extraction step, the material was collected by centrifugation at 2000× *g* for 10 min and the supernatants were discarded. Following the third acetone step, the samples were left to dry overnight in a fume hood.

### 4.3. Inoculum Preparation and Fungal Pretreatment

*Ganoderma lucidum* (GAN), *Pleurotus ostreatus* (PLE) and *Trametes versicolor* (TRA) were used as white-rot fungi (WRF) for biological pretreatments. As described elsewhere [72], morphological examination and molecular analysis targeting internally transcribed spacer (ITS) regions allowed the identification of the fungal species used in this study. Fungal inocula were prepared by culturing the individual WRF strains at 23 °C on 2.9% potato dextrose agar (PDA, Oxoid CM0139, Basingstoke, UK). After 10 days of growth, for each of the three WRF strains, inoculation disks (Ø = 10 mm) were taken from actively growing mycelium on the PDA plates and used to inoculate each sample of the lignocellulosic feedstocks (2 disks per sample), under solid state fermentation (SSF) conditions. To serve as solid media for WRF growth, the biomass was prepared as follows: approximately 1.5 g of previously dried and milled but not organic solvent-washed biomass was added to 5 mL deionised water and autoclaved in glass culture tubes capped with hydrophobic cotton. This was performed for each combination of the 3 WRF species and leaf or stem from the 3 acacia species. Additionally, non-inoculated biomass samples (non-WRF treated, NF controls) with an equal volume of deionised water added were included as negative controls. All cultures were incubated statically at 23 °C in the dark for 30 days, with a total of 48 duplicated samples: 3 acacia species × 2 organs (leaf or stem) × 4 treatments (3 WRF species plus control). WRF-pretreatment methodologies were adapted from procedures reported elsewhere [73,74]. After incubation, the inoculation disks were removed, 5 mL of deionised water was added, and the samples were thoroughly mixed and incubated at 30 °C for 24 h with constant mixing. Samples were then centrifuged (2000× *g* for 10 min), and supernatants were removed. The solid pretreated biomasses were washed twice with deionised water, dried at 60 °C, and stored for subsequent analyses and alkali pretreatment. For neutral sugars and lignin determinations, AIR samples were produced from this WRF-treated biomass (as described above).

### 4.4. Mild Alkali Pretreatment

A portion of the non-WRF treated (NF) and WRF-treated solid fractions (approximately 250 mg, dry weight) were subjected to a mild alkali treatment with 2.5 mL 0.1 M NaOH for 24 h at 150 rpm shaking at 21 °C. This step was performed with the aim of achieving biomass saponification and determining if combined WRF and mild alkali (WRF-ALK) pretreatments would act synergistically on the biomass, break ester-linkages, and release potentially valuable molecules. After the pretreatment, the samples were centrifuged (2000× *g* for 10 min), and the supernatants were discarded. The pretreated solids were washed 3 times in 5 mL of 0.025 M potassium acetate buffer (pH = 5.6) and twice with deionised water, dried at 60 °C, and stored for subsequent assays. For neutral sugars and lignin determinations, AIR samples were produced as described above from this alkali (ALK)-treated biomass. Furthermore, the alkali pretreatment was also employed on AIR samples prepared from non-WRF pretreated samples to assess the effect of 0.1 M NaOH on structural compounds. These samples are subsequently referred to as AIK.

### 4.5. Fourier-Transform Infrared Spectroscopy

Attenuated total reflectance Fourier transform mid-infrared (FTIR-ATR) spectroscopy was performed on all samples included in this study (AIR, AIK, WRF, ALK and WRF-ALK samples), as reported elsewhere [24]. Duplicate spectra were collected in the range 4000–400 cm^−1^ using a Bruker Optics Vertex 70 FTIR spectrometer purged by CO_2_-free dry air and equipped with a Brucker Platinum ATR single reflection diamond accessory. A Ge on KBr substrate beamsplitter and a liquid nitrogen-cooled wide-band mercury cadmium telluride (MCT) detector were used. Spectra were averaged over 32 scans at a resolution of 4 cm^−1^, and the 3-term Blackman-Harris apodization function was applied. The Bruker Opus 8.1 software (Bruker Optics GmbH, Ettlingen, Germany) was also used to: (i) remove eventual H_2_O and CO_2_ contributions and (ii) spectral smoothing using the Savitzky-Golay algorithm (window: 17 pt.). Absorbance spectra were converted to text files, imported into MatLab (v. R2021b; MathWorks, Natick, MA, USA) and averaged. Full spectra, or fingerprint region spectra (1800–800 cm^−1^), were vector normalised to unit length and the baseline was removed according to the automatic weighted least squares algorithm (polynomial order = 2) prior to statistical analysis using the Eigenvector PLS Toolbox (v. 9.0; Eigenvector Research, Wenatchee, WA, USA).

### 4.6. Saccharification

Non-pretreated, WRF-treated (30-day incubation with *G*. *lucidum*, *T*. *versicolor* or *P*. *ostreatus*) and mild alkali-treated biomass samples (ALK and WRF-ALK) were included in a saccharification assay, with four technical replicates for each sample, using an automatic platform as described elsewhere [75,76]. Briefly, 96-well plates containing biomass underwent saccharification analysis using a liquid handling platform (Tecan Evo 200; Tecan Group Ltd., Mannedorf, Switzerland) using enzymatic hydrolysis at 50 °C for 8 h. The enzyme cocktail contained commercially available Cellic CTec2 (Novozymes, Bagsvaerd, Denmark) in Na-Acetate buffer (25 mM; pH = 4.5) at 50 °C. The saccharification was performed in the liquid handling platform using a shaking Tecan reactor at an enzyme loading of 8 Filter Paper Units (FPU)/g. The reducing sugars released during hydrolysis were determined using a colorimetric assay involving 3-methyl-2-benzothiazolinone hydrozone (MTBH). Each plate contained standard reactions of 50 nmol, 100 nmol, and 150 nmol of glucose. Change in colour was read with a Tecan Sunrise microplate absorbance reader at 620 nm.

### 4.7. Neutral Monosaccharides

Acid hydrolysis and neutral monosaccharide determinations were performed as previously described [60], on non-pretreated, WRF-treated (30-day incubation with *P*. *ostreatus*) and mild alkali-treated (ALK and WRF-ALK) AIR samples. Briefly, 10 mg of each sample was weighed into 10 mL Pyrex glass tubes, and 100 μL H_2_SO_4_ (72% *w*/*w*) was added. Sealed tubes were left at 30 °C for 1 h. Samples were diluted to 4% H_2_SO_4_ (*w*/*w*) and autoclaved at 121 °C for 1 h. Once at room temperature, hydrolysates were neutralised using CaCO_3_, and the tubes were centrifuged (2000× *g* for 10 min) to obtain a clear supernatant. Carbohydrate separation and detection were achieved using high-performance anion exchange chromatography with pulsed amperometric detection (HPAEC-PAD). The ICS-5000 ion chromatography system (Dionex, Sunnyvale, CA, USA) was operated at 45 °C using a CarboPac SA10 column with a CarboPac SA10G guard column. An eluent generator prepared 1 mM KOH for 14 min isocratic elution at 1.5 mL min^−1^. Calibration standards (glucose, xylose and arabinose) were used for monosaccharide identification and quantitation.

### 4.8. Lignin Measurement

Acetyl bromide soluble lignin percentages were determined in duplicate for non-pretreated, WRF-treated (30-day incubation with *P*. *ostreatus*) and mild alkali-treated (ALK and WRF-ALK) AIR samples, as previously reported [77]. To approximately 10 mg of each sample, 500 μL of freshly prepared 25 % (*v*/*v*) acetyl bromide solution in glacial acetic acid was added, and the tubes were capped and left at 50 °C for a total of 3 h. Following lignin solubilisation, the tubes were cooled, and their contents were diluted by the addition of 2 mL of 2 M NaOH. A further addition of 350 μL of 0.5 M hydroxylamine hydrochloride to each tube ensured the decomposition of polybromide ions [78]. Final volumes were adjusted to 10 mL with glacial acetic acid and centrifuged (2000× *g* for 10 min) to produce particulate-free supernatants. From there, 200 μL of each sample was transferred to UV-transparent 96-well plates (UV-Star, Greiner Bio-One, Frickenhausen, Germany). Absorbance at 280 nm was measured with a plate reader (Perkin Elmer, Multimode Plate Reader 2300 EnSpire, Waltham, MA, USA). Blank negative controls were included, and their absorbance at 280 nm was set as the absorbance baseline. Lignin dry weight percentages were calculated according to Equation (1):

*ABSL*% = (*A*_280_/(*SAC* × *PL*)) × (*V_R_*/*W_S_*) × 100% (1))

where *ABSL*% is the acetyl bromide-soluble lignin percentage content; *A*_280_ is the absorption reading at 280 nm; *PL* is the pathlength determined for the 96-well microplates with a volume of 200 μL per well used during the analysis (0.556 cm); *V_R_* is the reaction volume (litres); *W_S_* is the sample weight (g); and *SAC* is the specific absorption coefficient of 23.0772 g^−1^ L cm^−1^, as reported elsewhere [79].

### 4.9. Statistical Analysis

All univariate descriptive statistics, analyses of variance and Tukey’s range tests were performed using Statistica (v. 8.0; StatSoft, Tulsa, OK, USA). For the *t*-tests on spectral data to unveil the underlying chemometric relationships between FTIR-ATR spectra, an R-based data analysis platform was used [80].

## 5. Conclusions

This study focused on the assessment of the saccharification and valorisation potential of invasive vegetation. Under our experimental conditions, an increase in saccharification potential was observed when mild alkali pretreatment was employed, and a potential synergistic effect occurs when combined with WRF pretreatments. Moreover, the compositional characterisation and pretreatment mechanisms contribute to a better understanding of the potential routes for the valorisation of *Acacia* spp., thus advancing the research into novel lignocellulosic crops. The utilisation of lignocellulose for more sustainable production of biofuels and other biomaterials depends on a deep understanding of the feedstock composition and the mechanisms of action of the employed processing methodologies. Given their vigorous and spontaneous growth, biomass from the studied invasive *Acacia* spp. often accumulates excessively in unmanaged agroforestry areas, thus exacerbating wildfire risks. Open pile burning in the vicinity of biomass generation is frequently the only economic disposal option. By identifying economic opportunities for invasive vegetation valorisation, landowners would have a monetary incentive to cut back excessive vegetation more frequently and employ more efficient land management practices. As excessive vegetation can exacerbate wildfire risks, improved land management would help to control the spread of spontaneous high biomass-producing invasive species, mitigating the wildfire risk, which is a serious threat in Mediterranean-type climates.

## Figures and Tables

**Table 1:** Assignment of relevant FTIR-ATR absorption bands characteristic of cell wall biomass from *Acacia* spp.

Region	Band (cm^−1^)	Assignment	Cell Wall Feature
*a*	**1728** [35,36,37]	C=O vibration	Xylan
*b*	**1640** [38,39,40]	COO- vibrations	Non-esterified carboxyl groups
*c*	**1612** [41,42,43]	Aromatic skeletal vibration	Lignin
*d*	**1314** [37,42,44]	Syringyl monomer vibration	Lignin
*e*	**1238** [37,45,46]	C-O vibrations of acetyl	Xylan
*f*	**1166** [37,47,48,49]	O-C-O asymmetric stretching (glycosidic link) all residues	Polysaccharides
*g*	**1055** [46,50,51]	C-O, C-C and O-C-H vibration	Cellulose
*h*	**1030** [50,51]	C-O, C-C and C-C-O stretching	Cellulose

**Table 2:** Saccharification of leaf and stem biomass (nmol mg^−1^) from *Acacia* spp. measured by a high-throughput saccharification assay after 8 h incubation in a hydrolytic enzyme mixture. Acronyms: AIR, alcohol insoluble residue; AIK, alcohol insoluble residue treated with a mild alkali pretreatment; NF, no fungi; GAN, *G. lucidum*; PLE, *P. ostreatus*; TRA, *T. versicolor*. Values are expressed as mean ± standard error.

	Leaf				Stem				
	**AIR**	**AIK**			**AIR**	**AIK**			
*A. dealbata*	125.5 ± 12.6	243.3 ± 7.7			197.9 ± 11.1	690.0 ± 16.7			
*A. longifolia*	51.7 ± 5.6	79.2 ± 6.6			80.9 ± 9.1	324.0 ± 15.5			
*A. melanoxylon*	81.8 ± 14.6	104.6 ± 12.4			195.6 ± 29.1	397.9 ± 71.2			
	**NF**	**GAN**	**PLE**	**TRA**	**NF**	**GAN**	**PLE**	**TRA**	**No alkali**
*A. dealbata*	140.0 ± 18.2	208.0 ± 15.5	194.1 ± 16.9	240.8 ± 40.4	243.9 ± 21.7	200.1 ± 18.2	276.9 ± 27.0	265.9 ± 26.9	
*A. longifolia*	115.1 ± 4.5	172.1 ± 12.8	172.1 ± 12.8	111.0 ± 16.2	230.7 ± 11.2	107.9 ± 5.3	162.5 ± 24.0	141.7 ± 2.6	
*A. melanoxylon*	160.8 ± 17.8	129.3 ± 9.7	149.6 ± 3.4	138.9 ± 16.9	207.6 ± 16.3	133.6 ± 2.9	234.2 ± 20.9	162.5 ± 6.1	
	**NF**	**GAN**	**PLE**	**TRA**	**NF**	**GAN**	**PLE**	**TRA**	**Alkali**
*A. dealbata*	243.4 ± 19.4	154.0 ± 10.8	179.6 ± 31.0	205.4 ± 38.8	608.1 ± 63.3	375.7 ± 7.5	702.3 ± 46.8	526.3 ± 80.5	
*A. longifolia*	105.2 ± 13.9	81.6 ± 5.2	94.7 ± 3.2	107.4 ± 8.5	349.0 ± 50.0	258.8 ± 28.0	289.1 ± 24.8	254.7 ± 42.1	
*A. melanoxylon*	166.7 ± 13.1	73.9 ± 10.9	70.6 ± 8.3	118.2 ± 18.9	236.4 ± 23.3	332.8 ± 52.4	369.8 ± 69.8	245.6 ± 17.1	

**Table 3:** Mean percentage (%) composition of alcohol insoluble residues (AIR) prepared from pretreated biomass from three *Acacia* spp. Pretreatment acronyms: NP, non-pretreated control samples; WRF, samples pretreated with *Pleurotus ostreatus*; ALK, alkali-pretreated samples; WRF + ALK, samples pretreated with *P*. *ostreatus* followed by the alkali pretreatment. Values are the mean ± standard error of the sample replicates.

**Leaf**					
	**Glucose**	**Xylose**	**Arabinose**	**Lignin**	
**NP**	14.5 ± 0.6	7.1 ± 0.9	2.7 ± 0.4	16.7 ± 0.4	*A. dealbata*
**WRF**	15.8 ± 0.2	7.4 ± 0.3	2.4 ± 0.2	14.6 ± 0.8	
**ALK**	19.3 ± 4.1	9.7 ± 1.6	2.8 ± 0.1	15.0 ± 0.7	
**WRF + ALK**	18.3 ± 1.4	8.1 ± 0.8	2.8 ± 0.2	12.4 ± 0.6	
**NP**	15.8 ± 1.0	6.6 ± 0.5	1.8 ± 0.3	13.8 ± 4.4	*A. longifolia*
**WRF**	14.3 ± 0.5	6.0 ± 0.8	1.6 ± 0.1	8.6 ± 0.7	
**ALK**	17.8 ± 0.4	6.3 ± 0.3	1.9 ± 0.2	9.8 ± 2.6	
**WRF + ALK**	17.5 ± 3.9	7.4 ± 1.8	2.0 ± 0.4	7.3 ± 0.2	
**NP**	18.3 ± 0.6	7.8 ± 0.5	3.3 ± 0.3	18.3 ± 1.6	*A. melanoxylon*
**WRF**	19.3 ± 1.2	8.3 ± 1.3	3.2 ± 0.2	15.1 ± 0.4	
**ALK**	20.4 ± 0.3	8.6 ± 0.5	3.4 ± 0.1	14.1 ± 2.4	
**WRF + ALK**	20.6 ± 0.7	8.4 ± 0.3	3.7 ± 0.0	12.3 ± 0.9	
**Stem**					
	**Glucose**	**Xylose**	**Arabinose**	**Lignin**	
**NP**	37.1 ± 0.4	16.4 ± 0.8	1.3 ± 0.1	19.2 ± 0.5	*A. dealbata*
**WRF**	39.7 ± 6.3	15.2 ± 0.1	1.1 ± 0.2	18.6 ± 0.6	
**ALK**	39.3 ± 1.7	15.3 ± 0.3	1.5 ± 0.1	17.4 ± 0.4	
**WRF + ALK**	43.1 ± 4.6	16.7 ± 1.5	1.3 ± 0.2	16.7 ± 0.6	
**NP**	42.3 ± 0.1	14.5 ± 0.5	1.2 ± 0.0	20.0 ± 0.2	*A. longifolia*
**WRF**	36.0 ± 2.7	13.8 ± 1.5	1.4 ± 0.3	18.2 ± 0.1	
**ALK**	40.0 ± 5.1	14.1 ± 1.2	1.5 ± 0.2	18.0 ± 0.5	
**WRF + ALK**	40.5 ± 0.9	14.7 ± 0.1	1.6 ± 0.1	18.2 ± 1.3	
**NP**	25.7 ± 0.3	11.8 ± 0.4	1.9 ± 0.0	19.7 ± 0.3	*A. melanoxylon*
**WRF**	29.9 ± 0.6	13.0 ± 1.3	1.9 ± 0.2	19.1 ± 0.1	
**ALK**	34.6 ± 3.6	14.7 ± 1.2	1.8 ± 0.3	16.8 ± 0.4	
**WRF + ALK**	32.4 ± 5.2	14.5 ± 2.4	1.9 ± 0.4	18.2 ± 0.4	

## Data Availability

The datasets generated during and/or analysed during the current study are available from the corresponding author on reasonable request.

## Supplementary Materials

The following supporting information can be downloaded at: https://www.mdpi.com/article/10.3390/molecules27207006/s1, Table S1. Percentage of recovered biomass after white-rot fungi (WRF; 30-day incubation) and mild alkaline 0.1 M NaOH for 24 h at 21 °C (ALK) pretreatments.

## Sample Availability

Samples of the compounds are not available from the authors.
