# Supplementary material for: Valorisation Potential of Invasive Acacia dealbata, A. longifolia and A. melanoxylon from Land Clearings
Source: Molecules. 2022 Oct 18;27(20):7006. doi: 10.3390/molecules27207006 (PMC9610895; doi:10.3390/molecules27207006)
Supplement: Supplementary file 1 [file molecules-27-07006-s001.zip › molecules-1951819-supplementary.pdf]

Supplementary Table S1. Percentage of recovered biomass after white-rot fungi (WRF) and mild alkaline 0.1M NaOH (ALK) pretreatments.

		**WRF*-pretreated**	**ALK-pretreated**	**WRF* + ALK pretreated**
**Leaf**	*Acacia dealbata*	94.3%	93.6%	91.7%
	Acacia longifolia	97.5%	96.8%	94.9%
	Acacia melanoxylon	90.5%	89.0%	84.8%
**Stem**	*Acacia dealbata*	96.5%	95.7%	93.2%
	Acacia longifolia	97.2%	96.5%	94.7%
	Acacia melanoxylon	95.3%	95.0%	94.2%

* Based on *Pleurotus ostreatus* pretreatment.
